# Hyaluronic acid concentration predictably modulates physicochemical properties and clinical performance in ophthalmic viscosurgical devices: integrated laboratory and prospective clinical assessment

**DOI:** 10.1186/s12886-026-04924-4

**Published:** 2026-05-20

**Authors:** Alejandro Melero, Josune Torrecilla, Ander Pino, José María Alonso, Daniel Coello, Sandra Sáez de Arregui, Gonzaga Garay-Aramburu, Silvia López-Plandolit, Olaia Guergué, Iker Henares, Amaia Huguet-Casquero, Miguel Ucelay López de Heredia, Raúl Pérez González

**Affiliations:** 1 i+Med S. Coop, Hermanos Elhuyar, 6, Vitoria-Gasteiz, Spain; 2 Araba University Hospital, Vitoria-Gasteiz, Spain; 3 Biobizkaia Health Research Institute; Osakidetza, Basurto University Hospital, Bilbao, Spain; 4 Faculty of Medicine, University of the Basque Country (EHU), Leioa, Spain; 5 Ophthalmology Research Network, Instituto de Salud Carlos III, Madrid, Spain

**Keywords:** Ophthalmic viscosurgical device, Hyaluronic acid, Cataract surgery, Rheology, Cohesive–dispersive index

## Abstract

**Background:**

Ophthalmic viscosurgical devices (OVDs) are essential in cataract surgery for maintaining anterior chamber stability and protecting intraocular tissues. This study evaluated the physicochemical and rheological properties of three hyaluronic acid–based OVDs (OpHLINE^®^ 1.4%, 2%, and 3%) and assessed their clinical performance.

**Methods:**

A two-phase investigation was conducted: (1) characterization of OpHLINE formulations versus four commercial comparators, including cohesive–dispersive analysis and viscosity–shear profiling; and (2) a prospective clinical trial in 68 patients undergoing phacoemulsification with intraocular lens implantation. Primary endpoint was workspace maintenance; secondary endpoints included ease of OVD removal, endothelial cell density (ECD) loss, and intraocular pressure (IOP) changes.

**Results:**

By varying HA concentration at constant molecular weight, we achieved direct control of rheological properties and CDI, generating distinct functional profiles: OpHLINE 1.4% behaved cohesively with rapid aspiration, OpHLINE 3% exhibited dispersive-like retention while maintaining space, and OpHLINE 2% combined cohesive handling with the highest shear-resistant viscosity. Clinically, OpHLINE 2% maintained an intermediate behaviour regarding space maintenance, ease of removal, ECD and IOP, while OpHLINE 3% provided the highest full chamber maintenance during all surgical steps. OpHLINE 1.4% enabled the easiest removal (95% adequate) and the lowest ECD loss (12.9%). IOP spikes ≥ 30 mmHg occurred in 13.6%, 20.8%, and 30.4% of eyes at 6 h for 1.4%, 2%, and 3%, respectively, resolving without sequelae.

**Conclusion:**

This study demonstrates that cohesive–dispersive behaviour and shear-dependent viscosity can be predictably tuned by HA concentration alone, enabling a modular, evidence-based approach to OVD selection. OpHLINE series provide evidence for three distinct behaviours: 1.4% showed optimal removal and endothelial protection; 2% offered balanced rheological and clinical properties; 3% provided the most consistent anterior chamber stability during surgery. These results allow surgeons to tailor intraoperative performance without altering polymer chain length.

**Trial registration:**

This study was retrospectively registered (OpHLINE-PIC01-2020), approved on 8 February 2021 by the Ethics Committee for Research with Medicines of Euskadi (CEIm-E, Spain, code PS2020065). Trial Registration: ClinicalTrials.gov, NCT ID Number NCT07343973, Registered on 07 January 2026.

**Supplementary Information:**

The online version contains supplementary material available at 10.1186/s12886-026-04924-4.

## Introduction

Cataract, the progressive opacification of the crystalline lens, remains the leading cause of blindness worldwide and its prevalence continues to rise with increasing life expectancy [1, 2]. Cataract surgery, primarily performed by phacoemulsification with intraocular lens implantation, offers excellent visual outcomes but remains technically demanding and dependent on anterior chamber stability [3, 4].

Ophthalmic viscosurgical devices (OVDs) are essential for space maintenance, endothelial protection, and controlled IOL implantation. They are classified as cohesive, dispersive, or viscoadaptive, each with distinct intraoperative advantages [5, 6]. Cohesive OVDs facilitate space maintenance and easy removal, dispersive OVDs provide sustained endothelial protection, and viscoadaptive OVDs exhibit variable behaviour depending on surgical forces.

Strategic OVD selection is critical in complex cataract surgery. Dense nuclei require higher phacoemulsification energy, increasing turbulence and endothelial risk [4]; dispersive OVDs offer superior protection, often combined with cohesive agents in the soft-shell technique [7]. The Tri-Soft Shell Technique further enhances safety by layering dispersive, cohesive, and viscoadaptive OVDs [8]. Other scenarios demand tailored use: dispersive OVDs for small pupils or compromised endothelium, cohesive agents for shallow chambers, and viscoadaptive OVDs when capsule rupture risk exists. Older patients frequently present multiple risk factors, reinforcing the value of dispersive or combined strategies [4]. Ultimately, OVD choice should reflect cataract morphology and ocular comorbidities.

OVD selection depends not only on surgical phase and ocular anatomy but also on rheological and physicochemical properties. Viscosity, elasticity, and pseudoplastic behaviour determine how an OVD maintains space, coats tissues, and resists aspiration. Higher viscosity and elasticity help preserve anterior chamber depth and protect tissues, while pseudoplasticity enables easy injection and aspiration yet stability at rest. Molecular weight and concentration shape the cohesive–dispersive balance, influencing chamber stability, endothelial protection, and ease of removal [9, 10]. Modern OVDs are primarily hyaluronic acid (HA) based, either as pure formulations or combined with components such as chondroitin sulphate to enhance dispersive properties and endothelial protection. Understanding these characteristics is essential for tailoring OVD performance to surgical demands, and ongoing innovations continue to refine their role in routine and complex cases.

The Healon™ series (Johnson & Johnson) includes cohesive agents (Healon PRO, Healon GV™) and the viscoadaptive Healon5™, which shifts between cohesive and dispersive behaviour under varying shear. Alcon offers DuoVisc^®^ (ProVisc^®^ cohesive + Viscoat^®^ dispersive, with chondroitin sulfate) and DisCoVisc^®^, a viscous dispersive OVD combining HA and chondroitin sulfate. Bausch + Lomb provides ClearVisc™ and StableVisc™, dispersive and cohesive agents supplemented with antioxidants (sorbitol). These products are widely used in advanced techniques such as the soft-shell and Tri-Soft Shell approaches. However, as emphasized by Arshinoff’s classification [6], their viscoadaptive behaviour and compositional complexity introduce confounding variables, making them unsuitable for controlled analysis of HA concentration at constant molecular weight.

This work analyses how controlled variation in HA concentration at constant molecular weight influences rheological properties and cohesive–dispersive behaviour and relates these differences to intraoperative performance. The study combines laboratory characterization with prospective clinical assessment to establish an evidence-based framework for OVD selection.

## Materials and methods

Four HA-only OVDs—Biolon, Biolon Prime, Microvisc, and BBTVisc—were included as comparators for analysis of physicochemical properties. OpHLINE, a newly developed HA-based OVD series, was designed to provide a systematic range of rheological behaviours by varying HA concentration at constant molecular weight, eliminating confounding factors from polymer chain length or additional components.

### Physicochemical and rheological characterization

#### Rheology

Rheological measurements were carried out using a rheometer (Discovery HR-1), using a plate geometry (40 mm) at a constant temperature of 25 °C of a sample of approximately 1.5 ml of product. The rheological analysis was performed on each commercial product in duplicate. TRIOS software was used for data acquisition and Sigmaplot 15.0 for graphical representation.

#### Injectability

To assess the injectability of the OpHLINE series, the extrusion force was measured using a PCE/FB200 dynamometer (PCE Instruments, Jupiter, FL, USA) integrated with a SAUTER TVM 5000N230N (Ker & Sohn GmbH, Balingen, Germany) motorised vertical stand operating at compression speed of 15 mm/min at room temperature. The syringe and cannula used for testing were those supplied by the manufacturer.

#### Cohesive-dispersive index

To carry out the cohesive-dispersive index (CDI) measurements of the OpHLINE, the procedure described in by Poyer et al. [11]. In brief, the material is aspirated through a 0.5 mm pipette tip for 2 s at 127, 254, 381, 508, 610 and 711 mm Hg vacuum levels. The weight of the material aspirated is then measured. Subsequently, the data obtained is plotted as the percentage of material aspirated against the corresponding vacuum level. The slope of the steepest part of this curve is calculated and used to determine the CDI. In essence, the CDI is equal to the slope at the steepest portion of this curve. The % aspirated is calculated as follows:

$$\eqalign{ & {\rm{\% Aspirated}} \cr & = \left( {100 - {\rm{Final\,weight}}/{\rm{Initial\,weight}}} \right) \times 100 \cr}$$

The CDI analysis was performed on each sample in triplicates with three measurement repetitions per sample. The average and statistical difference for each measurement replica was calculated using SPSS statistical package version 29.0. and represented using SigmaPlot 15.0.

### Clinical assessment

#### Study design

This study was designed as a post-market and prospective clinical trial and was carried out in two research centres: Araba University Hospital (Vitoria-Gasteiz, Spain) and Basurto University Hospital (Bilbao, Spain). This trial was reported following the Strengthening the Reporting of Observational Studies in Epidemiology (STROBE) Statement [12]. The study protocol (code OpHLINE-PIC01-2020), as well as the subject information sheet and informed consent form, were reviewed and approved on 8 February 2021 by the local Ethics Committee for Research with Medicines of Euskadi (CEIm-E, code PS2020065, Spain), in accordance with the international ethical standards from the revised World Medical Association Declaration of Helsinki amended in 2013. Clinical trial was submitted for registration at ClinicalTrials.gov, NCT ID Number NCT07343973, registered on 07 January 2026 following a retrospective registration method (after patient enrolment). This study was conducted following the guidelines stablished in UNE-EN ISO 14155:2021 (Clinical research on medical devices for humans. Good clinical practices) and CPMP/ICH-GCP-E6/135/95 (Guidance on Good Clinical Practice of the European Medicines Agency). This study adheres to CONSORT guidelines for reporting clinical trials.

#### Subject selection

Subjects provided written informed consent before entry into the study and were recruited from March 2021 and followed till September 2022. A preliminary assessment of each patient was carried out by an ophthalmologist at the baseline visit and the medical history was completed. Patients were included in the study if they signed the informed consent, met all the inclusion criteria and do not meet any exclusion criteria shown in Table 3.

#### Intervention

OpHLINE^®^ is a sterile, non-pyrogenic viscoelastic solution of highly purified, biofermented, linear, high molecular weight sodium hyaluronate in phosphate-buffered saline, which acts as a viscosurgical adjuvant. OpHLINE^®^ is a CE certified OVD and is indicated as an adjuvant during surgical procedures involving the anterior chamber of the eye, especially in cataract surgery, including lens extraction and intraocular lens insertion. When introduced into the anterior chamber of the eye during surgery, it maintains the depth and integrity of the chamber, facilitating surgery and the insertion of the intraocular lens and protecting intraocular tissues. Depending on the final sodium hyaluronate concentration, OpHLINE^®^ is presented in three models of 1.2mL prefilled syringes: OpHLINE 1.4%^®^ (14 mg/mL), OpHLINE 2%^®^ (20 mg/mL) or OpHLINE 3%^®^ (30 mg/mL). The recommended gauges for OVD application are 23G or 25G cannula.

Included patients were scheduled for the baseline visit and underwent the surgical intervention following the usual procedure of the centre with the aid of OpHLINE^®^. Six different surgeons (3 men and 3 women) participated in the surgical interventions. The following phacoemulsification platforms were used: Stelaris Elite (Bausch&lomb), Centurion vision system (Alcon), Veritas vision system (Johnson&Johnson). The fluidics settings during the quadrant removal were set at 300mmHg vacuum, aspiration flow rate of 40 cc/min and PIO 55mmHg. The phaco-technique used was divide-and-conquer and the usual coaxial irrigation and aspiration technique of the anterior chamber and capsular bag was employed for OVD removal. A total of 69 patients were randomly assigned to enter the study following a randomized allocation to treatment groups. Efficacy and safety outcomes were recorded during the surgical procedure and patients were followed for 90 days. The outcome assessors were not masked but the data processing of the clinical results was assessed by blinded researchers not involved in the intervention.

#### Outcome measures

##### Efficacy assessment

The primary efficacy outcome was the ability of the different OpHLINE^®^ models to maintain the workspace during the surgical procedure. This was based on the results reported by investigators through a subjective survey completed during different phases of the surgical intervention: capsulorhexis, hydrodissection, phacoemulsification, and intraocular lens (IOL) implantation. The ability to maintain the workspace was classified into four categories: full chamber, adequate workspace, shallow chamber, and flat. Full chamber and adequate workspace were considered as positive performance. Although the inclusion of intraoperative OCT would have strengthen the subjective assessment of investigators, this was not included in the usual clinical practice of the research centres, presenting a limitation of the study.

The secondary efficacy outcomes comprised the ease of OVD removal at the end of the surgical intervention following usual aspiration/irrigation technique. It was classified into three categories: easy, normal, or problematic removal. Easy and normal removal were considered as adequate. Although the inclusion of OVD removal time records would have strengthen the subjective assessment of investigators, this was not included in the usual clinical practice of the research centres, presenting a limitation of the study. In addition, the endothelial cell density was evaluated at baseline and at the end of the follow up period using non-invasive specular microscopy with the aim of assessing the surgery-related cell loss.

##### Safety assessment

The nature, onset, duration and severity of all adverse events, as well as any association of an adverse event related to the product according to specialist criteria, were assessed and registered at each visit. To evaluate the safety profile of the product, all complications and adverse events were recorded with an accountability scale. In addition, the safety of the surgical intervention was assessed by intraocular pressure (IOP) monitoring. IOP spike incidence (≥ 30mmHg) was assessed by Goldman applanation tonometry at 6 h, 24 h, 7 days, 30 days and 90 days post-surgery. The use of rescue medication was recorded daily in the patients´ case report form.

#### Statistical analysis

For statistical significance, an alpha risk of 0.05 (*p* < 0.05) was established, statistical power was set at 80%, a 95% confidence interval was established and follow-up losses of 10% were estimated. Likewise, a standard deviation of 10 points was assumed. Considering the above criteria, the sample size was set at 23 patients for each OpHLINE^®^ model to obtain at least 70% positive performance in space maintenance during surgery. Randomization between OpHLINE^®^ models was performed using a computerized randomization program at the research centres. Quantitative endpoints are mean, standard deviation and range. Qualitative endpoints are the number and percentage of each model, the number of patients and IOP peaks. Collected data were analyzed by statistical software (SPSS statistical package version 29.0. and R-Studio version 4.3.2). Initially, a descriptive analysis of the sample was performed considering the demographics and baseline clinical variables of patients. For the comparison between the values within each group, a two-tailed-p-value for repeated means was calculated. The Student’s t-test for related samples was used; in the case of not following a normal distribution (tested by Shapiro-Wilk test), the Wilcoxon test was used. For intergroup comparisons, the chi-square test and the one-way ANOVA test (normal distribution) or Kruskal-Wallis (no normal distribution) test was used for qualitative or continuous variables respectively.

## Results

### Physicochemical and rheological characterization

#### OVD characterization

Table 1 summarizes the HA concentration and molecular weight (MW) of the OVDs included in this study. The three OpHLINE^®^ formulations share the same MW (2.0–2.2 MDa) and differ only in HA content (1.4%, 2%, and 3%), providing a controlled model to isolate the effect of concentration. The comparator products present the following changes: Biolon and Biolon Prime contain 1.0% and 1.2% HA, respectively [13, 14], at approximately 3 MDa [6]; Microvisc 1% incorporates a higher MW polymer of about 5 MDa [15]; and BBTVisc 1.5% employs a lower MW of roughly 2.3 MDa [16]. These OVDs represent the conventional approach to achieving a desired functional profile; either adjusting the MW of HA or modifying both MW and %HA. In contrast, OpHLINE^®^ enables evaluation based solely on concentration, treating it as a single variable.

**Table 1:** Formulation and Product information of OVD

Product	%HA	Molecular Weight	Cohesive-Dispersive Behaviour	Osmolarity	pH	Refrigertion	Needle Gauge	Fill Size
OpHLINE 1.4%	1.4%	2.0-2.2 mDa	Cohesive (CDI = 48.6)	280–400 mOsm/kg	7.2–7.6	No	27G	1.2 mL
OpHLINE 2%	2.0%	2.0-2.2 mDa	Cohesive (CDI = 38.0)	280–400 mOsm/kg	7.2–7.6	No	25G	1.2 mL
OpHLINE 3%	3.0%	2.0-2.2 mDa	Dispersive (CDI = 21.0)	280–400 mOsm/kg	7.2–7.6	No	23G	1.2 mL
Biolon	1.0%	3.0 mDa	Cohesive	~ 280 mOsm/kg	6.8–7.6	Yes	27G	1.0 mL
Biolon Prime	1.2%	3.0 mDa	Cohesive	~ 280 mOsm/kg	6.8–7.6	Yes	27G	0.8 mL
Microvisc	1.0%	~ 5 mDa	Cohesive	310 mOsm/kg	6.8–7.6	No	27G	0.55/0.85/1 mL
BBTVisc	1.5%	~ 2.3 mDa	Dispersive	320 mOsm/kg	6.8–7.6	No	27G	0.55/0.85 mL

Cohesive–dispersive behaviour, a key determinant of aspiration dynamics, is well documented for the comparator OVDs: Biolon and Microvisc are described as cohesive as measured [6, 13–15], while BBTVisc is dispersive as described by manufacturer [16]. For OpHLINE, this information was not previously available and was therefore quantified using the cohesive–dispersive index (CDI) following the method of Poyer et al. [11]. The results revealed an evident concentration-dependent trend: OpHLINE 1.4% exhibited a CDI of approximately 49, OpHLINE 2% a CDI of about 38, and OpHLINE 3% a CDI near 21.

When these findings are considered alongside formulation data, an evident pattern emerges. High MW and low HA concentration, as in Microvisc, favour cohesive behaviour, whereas lower MW and moderate HA concentration, as in BBTVisc, align with dispersive properties. The 0,2% difference in HA concentration in Biolon and Biolon Prime does not seem to be sufficient to alter significantly the cohesive-dispersive behaviour of the hydrogel. Within OpHLINE, increasing HA concentration at constant MW progressively shifts the profile from cohesive to dispersive-like, demonstrating that HA concentration alone can modulate handling characteristics without altering polymer chain length.

Although CDI provides precise insight into aspiration dynamics, it does not capture how these materials respond exactly to the variable shear conditions encountered during surgery. To address this, we next examined the rheological properties of all tested OVDs across a clinically relevant shear-rate range.

#### Rheological properties and cohesive-dispersive behaviour

**Fig. 1:**
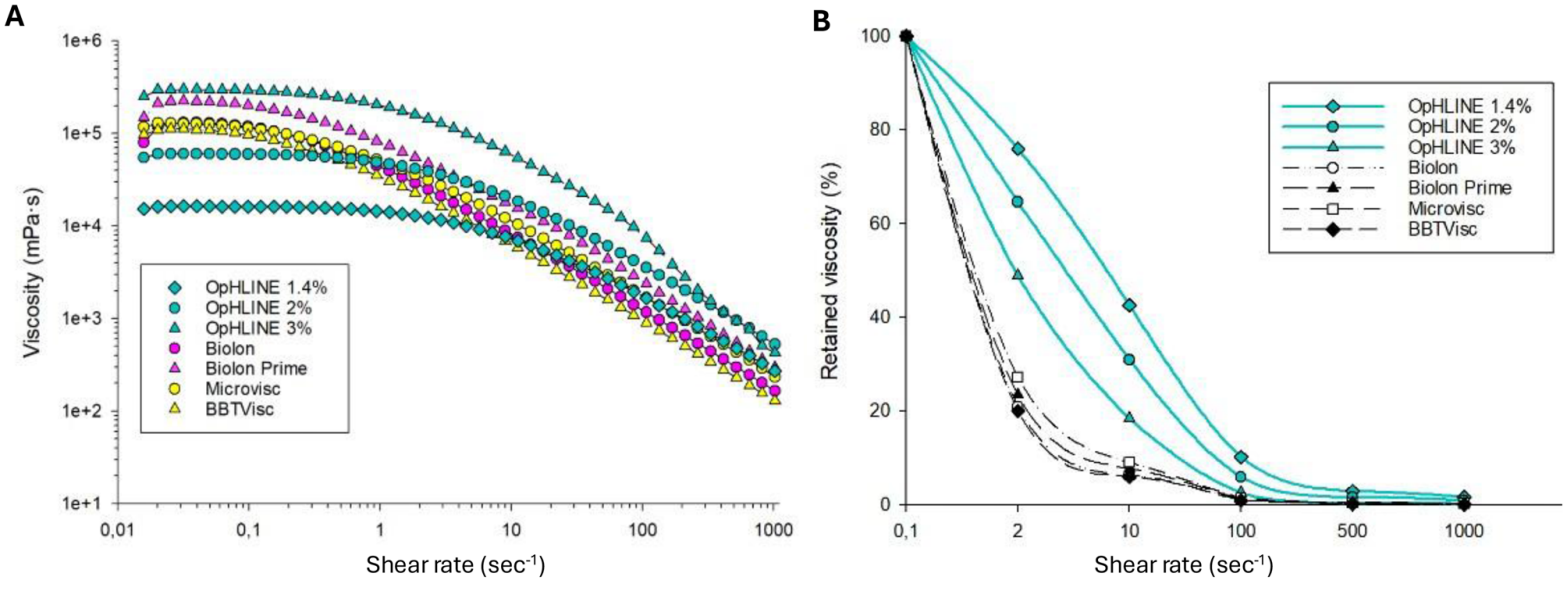
Rheological analysis. (**A**) Viscosity (mPa·s) versus shear rate (sec^− 1^) of tested OVD. (**B**) Decrease of zero shear viscosity (%) of tested OVD

Viscosity is a critical determinant of OVD performance during cataract surgery, influencing chamber stability, endothelial protection, and ease of removal [17]. High viscosity at low shear rates maintains anterior chamber depth and provides a protective coating during static phases, whereas lower viscosity under high shear facilitates injection and aspiration as surgical flow increases. Viscosity profiles of the seven OVDs were assessed across shear rates from 0.1 to 1000 s⁻¹ to simulate dynamic surgical conditions (Fig. 1A; Table 2). All products exhibited non-Newtonian, shear-thinning behaviour typical of HA-based viscoelastic agents, but the magnitude of viscosity reduction varied substantially among formulations.

**Table 2:** Viscosity values at surgery relevant shear rate values

Shear rate →	0.1 s^− 1^	2 s^− 1^		100 s^− 1^		1000 s^− 1^	
***OVD***	**Viscosity** **mPa·s**	**Viscosity** ** mPa·s**	**Retained viscosity (%)**	**Viscosity** ** mPa·s**	**Retained viscosity (%)**	**Viscosity** ** mPa·s**	**Retained viscosity (%)**
*OpHLINE 1.4%*	16252.3	12342.5	75.9	1660.1	10.2	272.5	1.7
*OpHLINE 2%*	59780.5	38668.0	64.7	3549.4	5.9	528.3	0.9
*OpHLINE 3%*	292389.0	143114.0	48.9	7453.6	2.5	427.3	0.1
*Biolon*	118358.0	24841.8	21.0	1170.8	1.0	166.4	0.1
*Biolon Prime*	200728.0	47064.9	23.4	2365.2	1.2	296.2	0.1
*Microvisc 1%*	115100.0	31360.0	27.2	1687.0	1.5	234.4	0.2
BBTVisc	96160.0	19280.0	20.0	895.9	0.9	129.6	0.1

The selected shear rates reflect clinical performance and experimentally standardized conditions [18]. A shear rate of 0.1 s⁻¹ is widely accepted as a proxy for zero-shear viscosity, representing the resting state of the OVD in the anterior chamber. A slow dynamic shear rate of 2 s⁻¹ corresponds to routine manoeuvres such as capsulorhexis and intraocular lens implantation [17, 19, 20]. For irrigation/aspiration, intermediate shear conditions occur—more energetic than routine steps but far below phacoemulsification extremes; a shear rate of about 100 s⁻¹ is therefore adopted as a practical mid-range proxy. Finally, 1000 s⁻¹ serves as a high-shear in vitro reference, approaching the turbulence generated during phacoemulsification, which is estimated to reach up to 10,000 s⁻¹ but remains challenging to replicate experimentally. This framework enables precise interpretation of rheological behaviour across the surgical continuum. 

Across all products, viscosity declined sharply as shear increased, but the rate of decline differed markedly (Fig. 1A). At 0.1 s⁻¹, viscosities were highest exceeding 200,000 mPa·s for Biolon Prime and approaching 300,000 mPa·s for OpHLINE 3%, with Biolon, Microvisc and BBTVisc right above or close to 100,000 mPa·s. At 2 s⁻¹, most comparators dropped to roughly 20–30% of their initial viscosity, whereas OpHLINE formulations retained viscosity proportionally to HA concentration: 76% for OpHLINE 1.4%, 65% for OpHLINE 2%, and 49% for OpHLINE 3%. This contrast is evident in the steep curves of Biolon, Biolon Prime, Microvisc and BBTVisc versus the more gradual decline of OpHLINE (Fig. 1B). By 100 s⁻¹, viscosity retention fell dramatically—10% for OpHLINE 1.4%, 6% for OpHLINE 2%, and 3% for OpHLINE 3%—while comparators dropped lower. At 1000 s⁻¹, nearly all products converged into a narrow band of 129–528 mPa·s, representing less than 1% of their original viscosity. These patterns highlight that gradual variations in HA concentration generate differences at low-shear performance, while at high-shear conditions minimize differences, thus HA concentration is key for a shear-resistant viscosity behaviour.

Taken together, our data suggest that cohesive–dispersive behaviour and intraoperative stability cannot be inferred from HA concentration or molecular weight in isolation. A combined analysis of shear-dependent viscosity and CDI is required to anticipate surgical performance. Within the OpHLINE series, we identified three shear-resistant viscosity ranges that remain stably separated throughout clinically relevant shear (≈ 0.1–100 s⁻¹) and tend to converge at extreme shear (≈ 1000 s⁻¹). In contrast, HA-only comparator OVDs that rely on higher HA% or higher MW (e.g., Biolon/Biolon Prime, Microvisc, BBTVisc) exhibit steep shear-thinning and minimal viscosity separation under increasing mechanical stress: their viscosity profiles become similar by the low-to-intermediate shear range and nearly overlap at 1000 s⁻¹, limiting true functional alternatives as surgical forces escalate. Thus, the OpHLINE viscosity stratification provides genuinely distinct options that persist where it matters clinically—under the shear conditions of capsulorhexis, hydrodissection, phacoemulsification, and I/A.

In addition, the injectability of OpHLINE series was assessed by monitoring the plunger-stopper break loose injection force over time including measurements of the initial glide force (PBF), peak force (Fmax) and dynamic glide force (DGF). Results showed mean PBF, DGF and Fmax values of 13.2 N, 15.4 N and 16.6 N for OpHLINE 1.4%, 13.9 N, 17.3 N and 19.3 N for OpHLINE 2% and 7.2 N, 20.4 N and 20.8 N for OpHLINE 3% respectively. As it could be observed, increasing HA concentration led to increased Fmax and DGF but injectability values remained between 11 and 25 N forces which is considered suitable for manual injection. The three models showed an initial force that triggered the movement of the plunger and the release of the product and subsequently, the required extraction force remained steady without peaks of maximum force. This highlights the homogeneous structure of the hydrogels with no impurities or bubbles that could interfere with the injection process thus indicating stable and predictable flow which is desirable for smooth injection.

The OpHLINE series demonstrated three rheologically distinct profiles—low, intermediate, and high viscosity—each maintaining shear resistance viscosity across clinically relevant ranges. High viscosity or sustained viscosity during surgery is particularly advantageous for preserving anterior chamber space [21, 22], an essential factor for intraoperative stability in cataract extraction. These differentiated rheological traits provide a rationale for evaluating whether such predicted behaviours translate into clinical performance under real surgical conditions.

### Clinical assessment

**Table 3:** Inclusion and exclusion criteria for study participation

Inclusion criteria	Exclusion criteria
− Patients over 18 years of age scheduled to undergo cataract removal by phacoemulsification followed by implantation of a posterior chamber intraocular lens in a single eye (operative eye). − Functional non-surgical eye, under investigator criteria.	− Ocular hypertension (IOP ≥ 22 mmHg). − Corneal endothelial damage (cellular count inferior to 2000/mm^2^). − Recurrent or chronic ocular inflammatory disease history. − Chronic or recurrent uveitis. − Acute ocular disease. − Internal or external infection. − Glaucoma or diabetic proliferative retinopathy. − Ocular trauma before surgery. − Congenital ocular anomaly or iris atrophy. − Any other pathology or physiological condition that could be worsened by cataract surgery. − Previous ocular surgery

Initially seventy-five patients were recruited in the study. Of these, four did not proceed to randomization, and two were randomized but did not undergo surgical intervention. Consequently, a total of 69 patients were ultimately enrolled and randomized in the study. Thirty-five patients were included in the Araba University Hospital and thirty-four were included in the Basurto University Hospital. During the monitoring visits, one patient was considered as a screening error because the exclusion criteria related to the baseline IOP had not been met (Table 3). Therefore, a total of 68 patients completed the follow up. Subject baseline demographics are shown in Table 4. A total of 22, 24 and 22 patients were treated with OpHLINE 1.4%^®^, OpHLINE 2%^®^ and OpHLINE 3%^®^, respectively. The mean age was 72 ± 6.6 years and the mean IOP was 15.68 ± 2.25 mmHg. During the surgical intervention, OpHLINE 1.4%^®^ was applied using 27G or 25G cannulas, over 68% and 32% of patients respectively. OpHLINE 2%^®^ and OpHLINE 3%^®^ were administered using a 25G cannula. Clinicians did not refer handling or procedural discomfort while the use of the OVDs.

**Table 4:** Baseline demographics of subjects included in the study

		Total	OpHLINE^®^1.4%	OpHLINE^®^2%	OpHLINE^®^3%
Sample size (*n*)		68	22	24	22
Average age (years)		72 ± 6.6	71.5 ± 7.4	72.9 ± 6.9	72.7 ± 5.7
Treated eye (frequency, %)	Right eye	28 (41%)	8 (36%)	13 (54%)	7 (32%)
	Left eye	40 (59%)	14 (64%)	11 (46%)	15 (68%)
Visual acuity at baseline		0.44 ± 0.16	0.46 ± 0.16	0.43 ± 0.15	0.43 ± 0.17
Intraocular pressure at baseline		15.68 ± 2.25	15.59 ± 2.38	15.58 ± 2.45	15.86 ± 1.96
Cellular density at baseline (cell/mm^2^)		2516 ± 264	2497 ± 218	2571 ± 266	2477 ± 302

#### Safety assessment

The population included for the safety analysis included all patients who underwent surgical intervention with the aid of OpHLINE^®^ (intention to treat, *n* = 69). The number of events that occurred was 23, being only one specifically related to OpHLINE^®^ as reported by investigator´s criteria. This single event was not considered a serios adverse event as it was related with injectability issues due to not following the IFU of the device regarding the tempering of the syringe prior to its clinical use. The rest of the events that were not specifically related to OpHLINE^®^, were considered common in his type of surgical interventions such as capsule rupture, endothelitis or iridian herniation (1%). These were resolved following usual clinical practice including vitrectomy or pharmacological treatment.

In addition, intraocular hypertension was the most described event (25%) and was treated with conventional hypotensive medication. In these sense, IOP spike incidence (≥ 30mmHg) was monitored during the follow up period (Table 5). IOP spikes were mainly reported after 6 h following the surgical intervention (22% incidence and mean IOP of 35 mmHg) with a single isolated case reported after 24 h (1% incidence and mean IOP of 32 mmHg). Although a higher HA concentration appeared to increase IOP spike incidence after 6 h, no statistical intergroup differences were found at any visit. No IOP spikes were reported during the rest of the follow up visits at 7 days, 30 days and 90 days.

**Table 5:** Intraocular pressure (IOP) spike incidence during the follow up visits

Follow up	OpHLINE^®^1.4% Nº of cases (%)	OpHLINE^®^2% Nº of cases (%)	OpHLINE^®^3% Nº of cases (%)
6 h	3 (13.64%)	5 (20.83%)	7 (30.43%)
24 h	0 (0%)	0 (0%)	1 (4.35%)
7 days	0 (0%)	0 (0%)	0 (0%)
30 days	0 (0%)	0 (0%)	0 (0%)
90 days	0 (0%)	0 (0%)	0 (0%)

#### Efficacy assessment

The clinical efficacy of OpHLINE^®^ was assessed by investigators as the ability of the OVD to maintain the workspace during the surgical procedure (Table 6). It could be observed that OpHLINE^®^3% showed the highest rates of full chamber maintenance during capsulorhexis, hydrodissection, phacoemulsification, and IOL implantation. In addition, all models showed a positive performance (full chamber or adequate workspace) over 80% of patients during surgery.

**Table 6:** Workspace maintenance ability of OpHLINE^®^ during the surgical procedure

Phase	Score	OpHLINE^®^ 1.4%	OpHLINE^®^ 2%	OpHLINE^®^ 3%
Capsulorhexis	Full chamber	8 (36%)	15 (62%)	15 (68%)
	Adequate workspace	10 (45%)	9 (38%)	5 (23%)
	Shallow chamber	4 (18%)	0 (0%)	2 (9.1%)
	Flat	0 (0%)	0 (0%)	0 (0%)
Hydrodissection	Full chamber	9 (41%)	15 (62%)	15 (68%)
	Adequate workspace	10 (45%)	9 (38%)	6 (27%)
	Shallow chamber	3 (14%)	0 (0%)	1 (4.5%)
	Flat	0 (0%)	0 (0%)	0 (0%)
Phacoemulsification	Full chamber	10 (45%)	15 (62%)	16 (73%)
	Adequate workspace	9 (41%)	9 (38%)	6 (27%)
	Shallow chamber	3 (14%)	0 (0%)	0 (0%)
	Flat	0 (0%)	0 (0%)	0 (0%)
IOL implantation	Full chamber	8 (36%)	16 (67%)	15 (68%)
	Adequate workspace	11 (50%)	8 (33%)	5 (23%)
	Shallow chamber	3 (14%)	0 (0%)	2 (9.1%)
	Flat	0 (0%)	0 (0%)	0 (0%)

When analysing the ease of OVD removal at the end of the surgery, OpHLINE^®^1.4% showed the best performance (adequate removal in 95% of patients) followed by OpHLINE^®^2% (adequate removal in 75% of patients) and OpHLINE^®^3% (adequate removal in 23% of patients) (Table 7). Regarding the endothelial cell density loss at the end of the follow up period, OpHLINE^®^1.4% showed the best performance (12.9% density loss) followed by OpHLINE^®^3% (19.5% density loss) and OpHLINE^®^2% (24.3% density loss) (Table 7).

**Table 7:** Efficacy of OVD removal and endothelial protection

Outcome	Phase	OpHLINE^®^ 1.4%	OpHLINE^®^ 2%	OpHLINE^®^ 3%
Adequate OVD removal	Post surgery	95%	75%	23%
Endothelial cell density loss	End of study	13%	24%	20%

As it has been stated, this is a post-market study of a CE marked medical device that has been commercialized for several years. Thus, post-market surveillance data is available regarding safety results of the product. Post-market user feedback provided additional insights into the product’s safety and usability profile, which aligned with and reinforced the findings from the clinical investigation. The safety analysis of the three OVD models (OpHLINE 1.4%, 2%, and 3%) revealed a very low incidence of adverse events, with an overall rate of just 0.004% based on post-market surveillance data Reported events —including intraocular pressure elevation, difficulty in product removal, and cannula detachment— were rare and non-serious. Additionally, feedback collected through user surveys reinforced these findings: 100% of respondents reported no adverse events, no complaints or suggestions, and expressed complete satisfaction with the product. All specialists confirmed that the available models were sufficient for their clinical needs and consistently used the product for the same indication.

## Discussion

Historically, commercially available OVDs have achieved functional differentiation through different features such as product composition, adjustments of HA molecular weight, modification of molecular weight distribution, combination formulations with distinct raw materials or cross-linking strategies. These approaches have been not adopted arbitrarily, but to optimize balance between injectability, ease of removal, IOP safety and endothelial protection.

However, in some cases the compositional complexity may introduce confounding variables making it difficult to establish cause-and-effect relationships between specific product properties and their viscoadaptive behaviour, rheological features and clinical performance. Rather than a replacement approach, this work provides a complementary strategy to conventional design of OVDs. Here it is analysed how controlled variation in HA concentration at constant molecular weight influences rheological properties and cohesive–dispersive behaviour and relates these differences to intraoperative performance. Hence, the aim of this study is not to underestimate established formulation paradigms which have been proven to optimally meet clinical needs, but to highlight the role of HA concentration as an isolated key factor in OVD design. However, it must be noted that concentration-only modulation might present inherent limitations when facing novel OVD designs as the rheological versatility of the final product could be hampered.

This study provides evidence that, at constant molecular weight, adjusting HA concentration alone predictably modifies the rheological and handling properties of OVDs, leading to clinically relevant differences during cataract surgery. By linking shear-dependent viscosity and cohesive–dispersive behaviour with intraoperative performance, these findings support an evidence-based approach to OVD selection grounded in measurable physical properties rather than empirical preference.

### Rheology as a predictor of surgical behavior

Our rheological analysis demonstrates that HA concentration and MW are not interchangeable parameters that would predict an OVD behaviour. Our analysis of Biolon and Biolon Prime showed only modest differences at zero shear and minimal divergence at high shear, indicating that a 0.2% HA increase at constant MW might have limited clinical relevance. Microvisc and BBTVisc followed a similar pattern: despite higher HA%, BBTVisc exhibited lower viscosity across all shear rates, confirming MW as the dominant determinant of viscoelastic properties. In contrast, the OpHLINE series revealed a clear concentration-driven stratification that persisted across clinically relevant shear rates. OpHLINE^®^ 1.4% maintained moderate zero-shear viscosity with proportionally greater viscosity retention under low-to-intermediate shear, supporting its cohesive profile and ease of removal. OpHLINE^®^ 2% profile integrates cohesive behaviour with intermediate viscosity across shear rates, supporting space maintenance and retaining the highest viscosity at 1000 s⁻¹ (≈ 528 mPa·s), thus suggesting a versatile option bridging viscosity with easy of removal. OpHLINE^®^ 3% reached ultraviscous levels comparable to viscoadaptive OVDs such as Healon 5 at surgery-relevant shear rates (2–100 s⁻¹) [23], where at zero-shear rate the viscosity of viscoadaptive OVDs ranges 7–18·10⁶ mPa·s, but drops to 10⁵ mPa·s at 1 s⁻¹, and 10³ mPa·s at 100 s⁻¹ [5, 6, 23, 24], thus converging with high-viscosity dispersive OpHLINE 3%. These findings confirm that HA concentration alone can produce clinically relevant differences in shear-dependent behaviour, allowing predictable modulation of space maintenance, endothelial protection, and aspiration dynamics without altering polymer chain length. Importantly, the similarity of OpHLINE^®^ 3% to viscoadaptive agents like Healon 5 within the 1–100 s⁻¹ range—where actual surgical forces occur—underscores its ability to deliver comparable chamber stability during critical steps.

### Clinical trial data on the OpHLINE^®^ series revealed distinct intraoperative performance across formulations

OpHLINE^®^ 3% showed the best performance regarding space maintenance during all surgical steps as full chamber was maintained over 68% of interventions, consistent with its rheological properties. These findings confirm that shear-resistant viscosity, rather than nominal viscosity alone, is the primary determinant of chamber stability under conditions of high aspiration and turbulence, as previously suggested in the literature [25].

### Clinical trial findings on the OpHLINE^®^ series confirmed a favourable safety profile across all formulations

The overall safety profile was favourable across all formulations although a slight trend of a concentration-dependent IOP spike trade-off was detected. Removal was viscosity-dependent: easiest with OpHLINE^®^ 1.4% and most demanding with OpHLINE^®^ 3%, though complete aspiration was achieved in all cases using standard techniques.

Transient IOP elevations were observed at 6 h in 13.6% (1.4%), 20.8% (2%), and 30.4% (3%) of eyes, with residual elevations at 24 h being rare (4.35% for 3%) and none persisting beyond 7 days. These results are consistent with previous reports of postoperative IOP spikes ranging from 13% to 35% within the first 4–8 h [26–32]. This phenomenon has been widely documented for decades and is generally considered temporary and benign [33]. Although the exact mechanism remains unclear, incomplete OVD removal and transient obstruction of the trabecular meshwork are recognized contributors [34]. Consequently, most authors recommend postoperative IOP monitoring and, when necessary, the use of antiglaucoma medication [25]. In this study, no clinically significant IOP elevations were detected beyond the immediate postoperative period. However, results indicate a trend of a concentration-dependent safety trade-off that should be considered when choosing the most appropriate model especially in high-risk population. This could be related to an increased obstruction of the trabecular meshwork that might interfere with an optimal product removal and consequently alter the natural flow of the aqueous humour through the Schlemm´s duct. In this sense the 30% IOP spike rate that was described at 6 h when using the OpHLINE^®^ 3% model, suggest that OVDs with high HA concentration should be carefully used or even avoided in patients predisposed to elevated IOP as a risk mitigation measure.

### Clinical trial data suggested the endothelial safety of the OpHLINE^®^ series

UNE-EN ISO 15798:2022 defines 500–1000 cells/mm² at 90 days as indicative of ocular damage. In this study, no patient reached ≤ 1000 cells/mm² at the end of the follow up; the mean endothelial cell density at 3 months was 2036 ± 494 cells/mm², well within normal limits despite percentage losses. These findings align with previous studies reporting losses of approximately 18–21% [35, 36], with final counts ranging from 1832 to 2310 cells/mm² [35, 37–39]. By contrast, the absence of an OVD has been associated with losses as high as 37–62% [40], underscoring the critical role of these devices in preserving corneal integrity. In this sense, OpHLINE^®^ 1.4% showed the lowest endothelial cell density loss at the end of the study (12.9%), reinforcing its suitability over the other models when endothelial safety is an important issue. However, in this study the record of baseline nuclear hardness, anterior chamber depth, axial length and presence of diabetes or other systemic comorbidities could have helped in a more robust attribution of postoperative endothelial loss protection to the OpHLINE^®^ series.

### Post-market surveillance

Real-world data from a broad user base corroborate these findings, reporting very low adverse event rates, primarily mild and related to transient IOP elevations or the need for more deliberate removal of higher-viscosity formulations. These observations emphasize the importance of thorough aspiration and early postoperative IOP monitoring.

### Practical framework for OVD selection

Varying HA concentration at constant molecular weight (2.0–2.2 MDa) generated distinct rheological and aspiration profiles that corresponded to different intraoperative behaviours. The 1.4% formulation combined high cohesivity with endothelial protection—challenging the traditional view that cohesive OVDs offer limited protection. This dual benefit, together with easy removal and minimal postoperative IOP fluctuations, makes OpHLINE^®^ 1.4% particularly valuable for cases prioritizing both safety and surgical efficiency. The 2% formulation combined cohesive handling with the greatest viscosity at high shear (≈ 528 mPa·s at 1000 s⁻¹), supporting stable anterior chamber conditions throughout surgery with intermediate endothelial protection, removal, and IOP control, making it a versatile option. The 3% formulation, despite sharing the same molecular weight, exhibited dispersive-like retention in CDI testing and provided enhanced stability during energy-intensive steps, challenging the assumption that dispersive behaviour depends on low molecular weight and viscosity [6, 17]. These results show that concentration-driven rheology can achieve dispersive retention without compromising space creation, reinforcing the need for classification frameworks based on measurable properties such as shear-dependent viscosity and CDI rather than molecular weight alone. In this context, the 3% formulation delivered the best space maintenance during all surgical phases.

These findings support an individualized approach to OVD selection based on rheology and surgical complexity. In energy-intensive cases such as hard or brunescent cataracts, where ultrasound energy and turbulence increase endothelial risk, OpHLINE 1.4% can help maintain chamber stability and provide sustained protection. When both volume and protection are priorities, combining this formulation with an intermediate option (OpHLINE 2%) enhances retention and stability without compromising safety. In eyes with deep anterior chambers or pseudoexfoliation syndrome, where controlled aspiration and structural support are critical, pairing a cohesive agent (OpHLINE 1.4%) with a dispersive-retentive formulation (OpHLINE 3%) optimizes depth and retention. These strategies illustrate how rheology-driven customization—rather than reliance on molecular weight—can improve intraoperative control and tissue protection across diverse scenarios, reinforcing the value of evidence-based OVD selection grounded in measurable physical properties.

## Electronic Supplementary Material

Below is the link to the electronic supplementary material.

Supplementary Material 1

## Data Availability

The datasets used and/or analysed during the current study are available from the corresponding author on reasonable request. All data generated or analysed during this study are included in this published article.

## Abbreviations

OVD: Ophthalmic viscosurgical device
HA: Hyaluronic acid
IOL: Intraocular lens
IFU: Instruction for use
IOP: Intraocular pressure
MW: Molecular weight
ECD: Endothelial cell density
CDI: Cohesive-Dispersive Index
CE: Conformité Européenne
STROBE: Strengthening the Reporting of Observational Studies in Epidemiology
